# The built environment and place attachment: Insights from Japanese cities

**DOI:** 10.1016/j.pmedr.2025.102969

**Published:** 2025-01-11

**Authors:** Jiuling Li, Mohammad Javad Koohsari, Andrew T. Kaczynski, Ryo Tanimoto, Reo Watanabe, Tomoki Nakaya, Yufeng Luo, Jing Zhao, Akitomo Yasunaga, Koichiro Oka, Tomoya Hanibuchi

**Affiliations:** a School of Advanced Science and Technology, Japan Advanced Institute of Science and Technology, Japan; b Faculty of Sport Sciences, Waseda University, Japan; c School of Exercise and Nutrition Sciences, Deakin University, Australia; d Department of Health Promotion Education and Behavior, Arnold School of Public Health, University of South Carolina, United States; e Faculty of Economics, Teikyo University, Japan; f Graduate School of Environmental Studies, Tohoku University, Japan; g School of Architecture and Urban Planning, Guangzhou University, China; h Faculty of Health Sciences, Aomori University of Health and Welfare, Japan; i Graduate School of Letters, Kyoto University, Japan

**Keywords:** Urban design science, Place bonding, Urban form, Science-based urban design, Walking, Asia, Local identity

## Abstract

**Objective:**

Place attachment plays an important role in individuals' health and well-being. Understanding the associations between urban design attributes and place attachment can inform strategies to promote place attachment. This study aims to examine the associations between walkable built environment metrics and place attachment.

**Methods:**

This study used data collected from October to November 2020, involving 25,340 adults across 21 major cities in Japan. Geographic address information was used to measure objective walkability, while perceived walkability and place attachment were assessed using adapted scales. Ordinary least squares regression models were employed to analyse associations.

**Results:**

For individual metrics, three objective measures—availability of destinations, population density, and street integration—were positively associated with place attachment. Several perceived measures, including access to shops, daily life facilities, public green spaces, public transports, the presence of paths, crime safety, and aesthetics, also showed positive associations with place attachment. For composite indices, traditional walkability, space syntax walkability, and perceived walkability were positively correlated with place attachment.

**Conclusions:**

These findings demonstrate the associations between walkable built environment metrics and place attachment, showing variations in metric types.

## Highlights

• Walkable built environments are linked to stronger place attachment.
• Destination availability and population density increase place attachment.
• Individual built environment factors and composite indices affect place attachment.

## 1 Introduction

Place attachment is closely related to health and well-being, as evidenced by several studies (Rollero and De Piccoli, 2010; Wiles et al., 2017). However, manipulating place attachment is complex and difficult (Pascual-de-Sans, 2004), because place attachment is a social construct that develops through social interactions and cultural consensus (Lewicka, 2011). Attempts to externally influence place attachment often face challenges, as it is shaped by deeply personal experiences and long-term interactions with place (Morgan, 2010). The built environment, which encompasses human-made surroundings where individuals live and work (Roof and Oleru, 2008), plays a role in fostering place attachment (Chang et al., 2023). Although modifying the built environment presents challenges in the short term, its long-lasting impact on people cannot be overlooked (Marteau et al., 2012). Several individual built environment metrics have been linked to place attachment (Chang et al., 2023; Sun et al., 2020). For example, a study conducted in China found that the height and volume of buildings, measured by the floor area ratio, were negatively correlated with place attachment (Chang et al., 2023). Another study discovered that diversity of neighbourhood facilities was positively linked to place attachment (Sun et al., 2020).

Nevertheless, this body of research is limited in several respects. First, it is necessary to use composite indices to consider the impact of the built environment on people. Individual built environment metrics do not exist in isolation, and their effects on residents should be considered collectively (Koohsari et al., 2020). However, there are only a few studies examining a composite index of neighbourhood walkability, including residential density, intersection density, and land use mix in relation to place attachment (Hanibuchi et al., 2012; van den Berg et al., 2024). For instance, a study from the Netherlands using structural equation models found a direct and positive link between objectively-measured neighbourhood walkability and place attachment (van den Berg et al., 2024). Individual built environment attributes also need attention, as understanding them can provide a more detailed insight into the relative impact of each attribute on individuals. Therefore, it is important to explore both individual and composite metrics of the built environment in relation to place attachment to obtain a comprehensive understanding of their impacts. Second, limited research has addressed the relationship between the built environment and place attachment in non-Western, densely populated, and culturally diverse urban areas (Sun et al., 2020).

To address these limitations, this study aimed to explore the associations between individual and composite metrics of neighbourhood walkability and place attachment, using a sample of Japanese urban dwellers.

## 2 Methods

### 2.1 Data source and participants

The data used in this study was sourced from the “Geo-social Survey for Urban Lifestyle Preferences” (GULP), which aims to compile a dataset with precise geographic location information to assist researchers in studying relationships between regions and individuals. Detailed information about the GULP procedures can be found elsewhere (Hanibuchi et al., 2022). Briefly, GULP consists of three separate surveys: metro survey, nonmetro survey, and nationwide postal survey. This study only used data from the metro survey, which was conducted online and covered 21 large cities in Japan, including Tokyo's special wards and government ordinance-designated cities. From October to November 2020, data were collected by a commissioned survey company (Nippon Research Centre Ltd). Participants who registered a group of panellists, referred to as the JAPAN Cloud Panel, were recruited for this survey. The survey employed quota sampling guided by a preset allocation table, ensuring that proportions of gender, age, and residence were consistent with those of the general population. Since the survey ended once the number of respondents for each quota was reached, calculating a response rate was not feasible. Finally, 30,000 participants were invited to complete the survey. Most of the participants agreed to provide detailed geographic address information for geographic analysis, including postal codes and house numbers. All participants who completed the survey were rewarded points equivalent to 46 yen and provided informed consent. The Ethics Committee of Human Subjects Research at the Graduate School of Engineering, Tohoku University, approved the survey (20A-10).

### 2.2 Measures

#### 2.2.1 Place attachment

Place attachment was measured through six items. Three items were adapted from well-established scales: “I am attached to this neighbourhood” (Kyle et al., 2005; Williams and Vaske, 2003), “My life is strongly tied to this neighbourhood” (Félonneau, 2004), and “In general, this neighbourhood has a good reputation” (Félonneau, 2004). Proxy measures, referring to outcomes or behaviours resulting from place attachment (e.g., being more willing to contribute to and improve the area) (Lewicka, 2011), were also utilised as indicators of attachment: “I want to improve this neighbourhood with my own hands” and “I want to preserve the culture and appearance of this neighbourhood” (Manzo and Perkins, 2006). The last item was newly created: “Then, what percentage of your neighbours do you think would answer ‘I am attached to this neighbourhood’ (strongly agree or somewhat agree)?”. All items were answered using a 5-point Likert scale. Responses for items 1 to 5 ranged from 1 (strongly agree) to 5 (strongly disagree), while item 6 was rated from 1 (80 % or more) to 5 (less than 20 %). Items were reverse-coded such those higher scores represented greater agreement and attachment, and scores for the six items were summed. Cronbach's alpha coefficient was used to evaluate the reliability of these six items measuring place attachment, yielding a result of 0.9.

#### 2.2.2 Walkable built environment metrics

Two types of objective and perceived measures of the built environment were included in this study. The objective walkable built environment metrics were assessed based on the geographic address information provided by participants. The longitude and latitude were first determined and matched to the census neighbourhoods, such as chocho-aza, a small neighbourhood with a size area mostly under 0.3 km^2^ and roughly equivalent to a block group in the United States census (Nakaya et al., 2014). In this study, a 1000 m network buffer zone spreading outwards from the chocho-aza centre was used to identify walkability metrics. Using the centroid of chocho-aza as the network buffer centre, rather than exact participant locations, helps protect data privacy while approximating local environments in this study. This approach is further supported by findings from another study in Japan on the built environment and walking, which reported minimal differences between using proximate locations, such as postal codes, and exact participant addresses (Tanimoto et al., 2023). We selected 1000 m for buffer distance as it typically represents the average walking distance for adults to a destination (Berke et al., 2007). However, some address information was not precise enough to target accurate chocho-aza, which would be inappropriate for using chocho-aza census variables. Therefore, only samples with address information that could be identified at chocho-aza level were used.

**Fig. 1:**
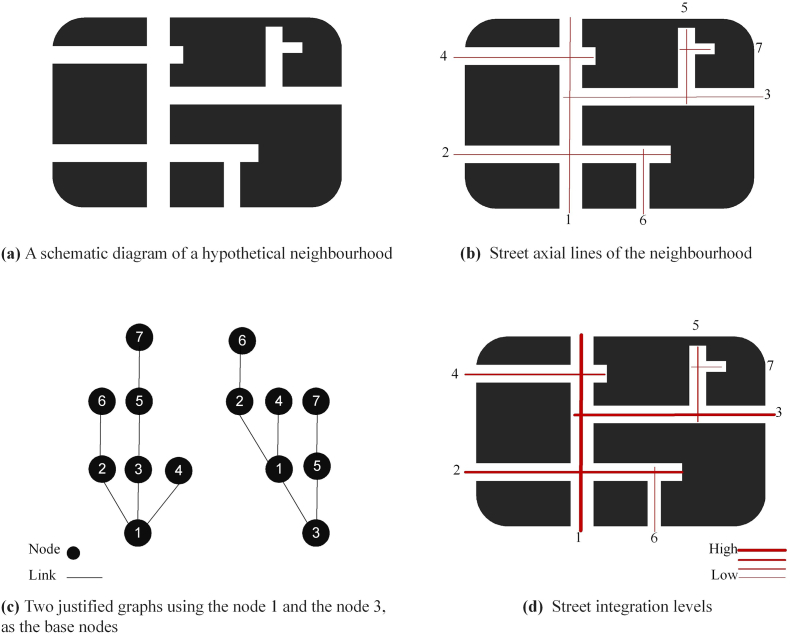
The concept of space syntax street integration.

Objective built environment measures include four individual attributes (availability of destinations, intersection density, population density, and street integration) and two composite indices (traditional walkability index and space syntax walkability). The availability of destinations was obtained by counting the number of different types of destinations within each 1000 m buffer of chocho-aza. A maximum of 18 types of destinations were considered, including coffee shops, laundries, convenience stores, gyms, parks, and so on. The details of the 18 types have been described elsewhere (Tanimoto et al., 2023). Intersection density was measured as the number of intersections divided by the land area of the buffer of chocho-aza using Japan road network data from ArcGIS Geo Suite provided by ESRI Japan. The road network data contains “nodes”, which are point data to represent the end of road segments. The number of intersections was obtained by counting the nodes having three or more connecting ways. Population density was determined from the 2020 Japanese census data. To measure street integration, the formula used was the number of nodes divided by the mean depth (Hillier, 2007), which represents the difficulty of reaching a specific location (Edgü and Ünlü, 2003). Fig. 1 shows the concept of space syntax street integration. In this study, angular analysis was used to calculate the mean depth, considering the angles people need to take while walking, rather than simply counting the number of turns (Turner, 2001; Watanabe et al., 2023). This is more relevant for analysing the integration of complex street patterns (Turner, 2001; Watanabe et al., 2023). Using the Space Syntax Toolkit and Depthmap X software developed by University College London (Turner, 2004), street integration values were calculated for all street segments across Japan, as well as street integration averages for each buffer of chocho-aza. Two composite walkability indices were calculated based on individual attributes. The first traditional walkability index consisted of destination availability, intersection density, and population density, and was adapted from Frank et al.'s (2010) walkability index, excluding retail floor area ratio to suit the Japanese context (Tanimoto et al., 2023). The traditional walkability index was computed using the following formula:

$$\text{Traditional walkability index}=\frac{z\;\left(\text{availability of destinations}\right)+z\;\left(\text{intersection density}\right)+z\;\left(\text{population density}\right)}{3}$$

Another composite index, space syntax walkability, differs conceptually from the traditional walkability index (Koohsari et al., 2016). Space syntax walkability was developed based on the concepts of space syntax, explaining walkability through street layout characteristics (Koohsari et al., 2016). The data required to calculate space syntax walkability are not difficult to obtain, as they only include population density and street integration. It was derived using the following formula (Koohsari et al., 2016):

$$\text{Space syntax walkability}=z\left[z\left(\text{population density}\right)\right)+2\times \left(z\left(\text{street integration}\right)\right]$$

It should be noted that in both of the aforementioned formulas, z indicates that normalised individual attributes were used in the calculations.

Perceived measures were assessed through several individual items that were directly asked in the metro survey of GULP. Items were adapted from the “Japanese version of the International Physical Activity Questionnaire Environmental Module” and the “Abbreviated Neighbourhood Environment Walkability Scale Japanese version”, and include: (1) access to shops; (2) access to daily life facilities; (3) access to public green spaces; (4) access to public transports; (5) presence of paths; (6) traffic safety; (7) crime safety; and (8) aesthetics. Participants responded to each item using a 5-point scale from 1 (many) to 5 (none) by following the question: “Do you have the following in your area (or neighbourhood)? *Within a 10-minute walk from your home.” Items from 1 to 5, as well as item 8, were reverse coded. All the items were also summed up to represent the composite perceived walkable environment with a higher composite score indicating a better perception. Cronbach's alpha coefficient of these items was 0.7.

#### 2.2.3 Covariates

Based on the previous study (Hanibuchi et al., 2012), several sociodemographic variables were included as covariates in the current analyses, including age, gender, marital status, education level, gross annual household income, and employment status. Additionally, due to the potentially close relationship between the length of residence and the formation of place attachment (Cheng et al., 2024), residence time at the current address was also considered as a covariate.

### 2.3 Statistical analysis

Descriptive analysis was used to compute the mean and standard deviation, or number and rate (%) of participants' characteristics and objective walkability metrics. Ordinary least squares regression models were employed to analyse the associations between walkability metrics and place attachment. Each walkability metric was tested in a separate model. For each analysis, unstandardised regression coefficients (*B*) and 95 % confidence intervals (95 % CIs) were reported. All analyses were conducted using STATA version 15.1, with a significance set at *p* < .05.

## 3 Results

### 3.1 Characteristics of study participants

After excluding participants who selected “I don't know” for education level and gross annual household income, as well as those with inaccurate address information, data from 25,340 participants were analysed. Table 1 describes the characteristics of the participants. Most participants had received higher education (81.5 %) and were employed (76.4 %). Additionally, over half of the participants were married (57.4 %) and had a household income of over 5 million yen per year (52.9 %). Overall, the majority of participants resided in the area for 5 to less than 20 years (38.1 %). The average score for place attachment was 19.9 (SD = 4.8). While some demographic differences were noted between the final sample and the original 30,000 participants, the overall distributions remained relatively stable. A comparison with the Japanese Census data for these 21 cities shows that the distributions of most demographic variables, including age, gender, and employment status, were similar (Statistics Bureau of Japan, 2020). However, education levels were notably higher in the study sample compared to the census data, with 81.5 % of participants reporting tertiary education or higher, compared to 61.5 % in the census. Table 2 presents the data on participants' walkable built environment metrics. The average number of destinations within the 1000 m buffer zone was 15.4 (SD = 2.6). Moreover, there were 241.3 (SD = 76.5) intersections per km^2^ and a population density of 13,580.6 (SD = 6457.3) people per km^2^. For street integration, the mean value was 215.8 (SD = 84.7). Access to public transports got the highest score, which was 4.0 (SD = 0.9) among all other individual perceptual metrics.

**Table 1:** Characteristics of participants from 21 large cities in Japan, 2020.

Variable	Study sample (*n* = 25,340) N (%) or Mean (SD)	Original sample (*n* = 30,000) N (%) or Mean (SD)	Population Census (*n* = 23,378,992) N (%)
Age			
20–64 years adults	23,589 (93.1)	27,883 (92.9)	21,354,704 (91.3)
65–69 years older adults	1751 (6.9)	2117 (7.1)	2,024,288 (8.7)

Gender			
Male	13,051 (51.5)	15,073 (50.2)	11,648,268 (49.8)
Female	12,289 (48.5)	14,927 (49.8)	11,730,724 (50.2)

Education level			
Below tertiary	4700 (18.5)	5646 (18.9)	6,791,570 (38.5)
Tertiary or higher	20,640 (81.5)	24,226 (81.1)	10,830,266 (61.5)

Employment status			
Employed	19,356 (76.4)	22,376 (74.6)	14,925,572 (78.3)
Unemployed	5984 (23.6)	7624 (25.4)	4,131,453 (21.7)

Marital status			
Single	10,806 (42.6)	13,205 (44.0)	8,544,568 (39.8)
Married	14,534 (57.4)	16,795 (56.0)	12,909,172 (60.2)

Gross annual household income			
<¥5,000,000	11,927 (47.1)	12,522 (47.6)	–
≥¥5,000,000	13,413 (52.9)	13,808 (52.4)	–

Length of residence			
Less than 5 years	7934 (31.3)	9073 (30.2)	5,850,479 (30.7)
5 to less than 20 years	9649 (38.1)	11,286 (37.6)	7,482,603 (39.3)
20 years or more	7757 (30.6)	9641 (32.1)	5,728,874 (30.1)

Place attachment	19.9 (4.8)	19.9 (4.8)	–

**Table 2:** Characteristics of walkable built environment metrics from 21 large cities in Japan, 2020 (*N* = 25,340).

Metrics	Mean (SD)
Individual walkability	
Objective attributes	
Availability of destinations (numbers)	15.4 (2.6)
Intersection density (intersections/km^2^)	241.3 (76.5)
Population density (people/km^2^)	13,580.6 (6457.3)
Street integration	215.8 (84.7)
	
Perceived attributes	
Access to shops	3.9 (1.0)
Access to daily life facilities	3.9 (0.9)
Access to public green spaces	3.9 (0.9)
Access to public transports	4.0 (0.9)
Presence of paths	3.8 (1.0)
Traffic safety	2.5 (0.9)
Crime safety	2.7 (0.9)
Aesthetics	3.1 (1.0)
	
Composite walkability	
Walkability index	0.0 (0.8)
Space syntax walkability	0.0 (1.0)
Perceived walkability	27.8 (4.0)

### 3.2 Associations between walkable built environment metrics and place attachment

After adjusting for covariates, Table 3 presents the associations between walkable built environment metrics and place attachment. Among objective metrics, availability of destinations, population density, and street integration were each positively associated with place attachment (*B* = 0.15, 95 % CI = 0.13–0.17, *B* = 0.05, 95 % CI = 0.04–0.05, and *B* = 0.01, 95 % CI = 0.01–0.02, respectively). Significant positive associations were observed between several perceived metrics — access to shops, daily life facilities, public green spaces, public transports, paths, crime safety, and aesthetics — and place attachment. Composite indices, including traditional walkability, space syntax walkability, and perceived walkability, were likewise positively associated with place attachment.

**Table 3:** Associations between walkable built environment metrics and place attachment from 21 large cities in Japan, 2020 (*N* = 25,340).

Metrics	Place attachment		
	*B*	95 % CI	
Individual walkability			
Objective attributes			
Availability of destinations	0.15 **	0.13	0.17
Intersection density	0.01	−0.00	0.01
Population density	0.05 **	0.04	0.05
Street integration	0.01 **	0.01	0.02
			
Perceived attributes			
Access to shops	1.29 **	1.23	1.34
Access to daily life facilities	1.59 **	1.52	1.65
Access to public green spaces	1.60 **	1.54	1.66
Access to public transports	1.52 **	1.46	1.59
Presence of paths	1.62 **	1.56	1.67
Traffic safety	0.04	−0.02	0.10
Crime safety	0.32 **	0.25	0.38
Aesthetics	2.29 **	2.24	2.35

Composite walkability			
Traditional walkability	0.35 **	0.28	0.42
Space syntax walkability	0.19 **	0.13	0.25
Perceived walkability	0.56 **	0.55	0.57

*B* = unstandardised regression coefficient; 95 % CI = 95 % confidence interval. Each walkability metric was examined separately, and adjusted for age, gender, education level, marital status, gross annual household income, employment status, and length of residence.

**p < .01.

## 4 Discussion

This study explored the association between several individual and composite metrics of walkability and place attachment. We found that several walkable urban design metrics were positively associated with place attachment, consistent with studies indicating that living in highly walkable areas results in stronger place attachment (Cheng et al., 2024; van den Berg et al., 2024).

As for objective individual measures, we found that the availability of destinations is linked to greater place attachment. This finding aligns with a previous study showing that public facilities positively correlate with cognitive attachment, a dimension of place attachment linked to memories and familiarity (Widya et al., 2019). Diverse destinations serve as social spaces, increasing opportunities for social interaction (Brown and Lombard, 2014). This interaction fosters social connections, which can strengthen place attachment (Raymond et al., 2010). Additionally, a diverse range of destinations can meet people's varied needs, which may foster functional attachment to the place (Moulay et al., 2018). Population density also showed a positive association with place attachment. However, some previous studies have shown that places with low population density often have stronger place attachment (Buffel et al., 2014; Ma, 2021). Higher population density tends to lower neighbourhood satisfaction (Sharp, 2019) and reduce social capital (Koohsari et al., 2021), both of which are highly correlated with place attachment (Lee and Jeong, 2021). Nevertheless, there may be another perspective proposed: dense areas can increase opportunities for people to interact, and such social contact may increase place attachment (Taima and Asami, 2019; Weijs-Perrée et al., 2017). Especially in the context of Japan, urban areas are more densely populated and larger, increasing opportunities for social interaction (Taima and Asami, 2019). For the two attributes related to street layouts, we found that intersection density showed no significant association with place attachment, while street integration was significant. This may be because these two measures of street layout focus on distinctive aspects. Intersection density refers to the physical connectivity of streets, while street integration is measured based on the topology concept, which takes into account visual connectivity (Hillier and Iida, 2005). Visual connectivity may effectively help people comprehend the image of a place (Jamil and Mahdzar, 2022), potentially increasing attention to space and enhancing place attachment by improving its legibility (Ujang and Shamsudin, 2012).

Our study also found significant associations between perceived built environment metrics and place attachment. Perceived and objective measures of the built environment are often not entirely aligned, which may result in differences in their associations with place attachment (Koohsari et al., 2021). For example, van den Berg et al., 2024 found that perceived walkability was an effective determinant of place attachment than objective walkability. Previous studies have indicated that the perceived accessibility of destinations such as shops, parks, daily life facilities, and public transportation enhances residents' abilities to meet their daily needs (Chan and Li, 2022; Pfeiffer et al., 2020). This convenience may increase functional satisfaction with the neighbourhood supporting place attachment (Chan and Li, 2022). This satisfaction is also reflected in the length of residence, a supporting factor for place attachment (Koohsari et al., 2023). Consistent with prior research (Chan and Li, 2022), our findings also indicate that the presence of paths positively supports place attachment. Paths encourage walking, which fosters emotional connections with the neighbourhood and enhances attachment (van den Berg et al., 2024). In this context, paths provide a foundation for experiencing and engaging in community interactions. Additionally, a safe and visually appealing environment can promote residents' sense of security and comfort, making them more likely to reside long-term and form emotional attachment (Leverentz et al., 2018). Our findings align with this, demonstrating that perceived safety and aesthetics are positively correlated with place attachment. Further studies are needed to explore these associations in different cultural, and urban contexts to better understand the mechanisms underlying these relationships.

In this study, we tested several composite walkability measures, both objective and perceived, in relation to place attachment. Testing composite measures is important as these metrics provide a more realistic view of the environment in which residents live. Environmental characteristics, such as access to destinations, population density, and street connectivity, naturally coexist and interact, and composite measures reflect this complexity better than individual metrics alone (Koohsari et al., 2020). Additionally, some composite measures, like space syntax walkability, are easier to calculate than individual items, facilitating study replicability and making the metrics more accessible for policymakers and urban designers (Koohsari et al., 2016). Our findings also indicated that perceived composite walkability was positively associated with place attachment. These perceived qualities are important, as they offer a practical approach to fostering place attachment when physical changes to the built environment may not be feasible. By focusing on perceived aspects such as safety, aesthetics, and accessibility, urban designers can enhance residents' connections to their neighbourhoods in ways that complement objective improvements to walkability (van den Berg et al., 2024).

Several limitations exist in the current study. The buffer zone defined in this study is likely to differ from the area where participants walk. Future studies can utilise more advanced technologies, such as global positioning systems, to accurately track the movements of people. This study used cross-sectional data and therefore cannot demonstrate a causal relationship between walkability metrics and place attachment. Despite these limitations, the strengths of this study include examining a diverse array of walkability metrics and using data covering 21 major cities throughout Japan.

## 5 Conclusions

Place attachment supports health and well-being, and urban design science informs how the built environment promotes it. This study highlighted the importance of considering both individual built environment attributes and composite indices of neighbourhood walkability in relation to place attachment. Further research is needed to explore these associations in diverse geographical contexts.

## Funding

MJK is supported by the Japan Society for the Promotion of Science (JSPS) Grants-in-Aid for Scientific Research (KAKENHI) (grant JP23K09701). TN was supported by the JSPS KAKENHI (grant JP20H00040). KO is supported by the JSPS KAKENHI (grant JP20H04113). TH was supported by the 10.13039/501100001691JSPS KAKENHI (grant JP17H00947, JP18KK0371, and JP24K00176).

## CRediT authorship contribution statement

**Jiuling Li:** Writing – original draft, Software, Methodology, Formal analysis, Conceptualization. **Mohammad Javad Koohsari:** Writing – review & editing, Supervision, Methodology, Funding acquisition, Conceptualization. **Andrew T. Kaczynski:** Writing – review & editing. **Ryo Tanimoto:** Writing – review & editing. **Reo Watanabe:** Writing – review & editing. **Tomoki Nakaya:** Writing – review & editing. **Yufeng Luo:** Writing – review & editing. **Jing Zhao:** Writing – review & editing. **Akitomo Yasunaga:** Writing – review & editing. **Koichiro Oka:** Writing – review & editing. **Tomoya Hanibuchi:** Writing – review & editing, Supervision, Investigation, Data curation, Conceptualization.

## Declaration of competing interest

The authors declare that they have no known competing financial interests or personal relationships that could have appeared to influence the work reported in this paper. In particular, none of the authors has a financial interest in the Space Syntax Limited company.

## Data Availability

Data will be made available on request.
